# Compact conformal tattoo-polymer antenna for on-body wireless power transfer

**DOI:** 10.1038/s41598-023-36335-6

**Published:** 2023-06-15

**Authors:** Xi Liang Chang, Pei Song Chee, Eng Hock Lim

**Affiliations:** 1 Department of Mechatronics and Biomedical Engineering, Universiti Tunku Abdul Rahman, 43000 Kajang, Malaysia; 2 Department of Electrical and Electronic Engineering, Universiti Tunku Abdul Rahman, 43000 Kajang, Malaysia

**Keywords:** Energy harvesting, Soft materials

## Abstract

This paper presents a 35.0 × 35.0 × 2.7 mm^3^ compact, low-profile, and lightweight wearable antenna for on-body wireless power transfer. The proposed antenna can be easily printed on a piece of flexible tattoo paper and transformed onto a PDMS substrate, making the entire antenna structure conform to the human body for achieving a better user experience. Here, a layer of frequency selective surface (FSS) is inserted in between the antenna and human tissue, which has successfully reduced the loading effects of the tissue, with 13.8 dB improvement on the antenna gain. Also, the operating frequency of the rectenna is not affected much by deformation. To maximize the RF-DC conversion efficiency, a matching loop, a matching stub, and two coupled lines are integrated with the antenna for tuning the rectenna so that a wide bandwidth (~ 24%) can be achieved without the use of any external matching networks. Measurement results show that the proposed rectenna can achieve a maximum conversion efficiency of 59.0% with an input power of 5.75 μW/cm^2^ and can even exceed 40% for a low input power of 1.0 μW/cm^2^ with a 20 kΩ resistive load, while many other reported rectennas can only achieve a high PCE at a high power density level, which is not always practical for a wearable antenna.

## Introduction

Wearable electronics have attracted much interest in recent years due to their wide applications in our daily life. They can be applied on many places such as smartwatches, smart clothing, and real-time health monitoring devices^1^. However, one main limiting factor of most commercialized wearable electronics is their power supplies^2^. Most of these electronic devices are battery-operated, but unfortunately, the battery itself has limited lifespan and its bulk size does not scale down as fast as electronics^3^. Nowadays, with the rapid development of 5G technology, where beamforming techniques are massively used, microwave wireless power transfer (WPT) has become an attractive solution to resolve the power charging problem^4^.

Flexible microwave antennas that are made of fabric materials have been broadly reported for wearable applications in recent years^5,6^. Wearable antenna also can be made by electroplating thin metal foils such as copper^7^ and gold^8^ on elastic dielectric substrates as well as inkjet-printing conductive nanoparticle inks^9^ onto flexible substrates. Soft fabrics are chosen for wearable antennas due to their good conformality, flexibility, and low cost^10^. Despite these excellent features, woven fabric may have a dielectric loss as high as 8.5 dB/m^11^. Also, gain reduction has been observed in the embroidery antenna reported in Ref.^5^ due to the higher resistance of the conductive yarn. Inkjet-printed antennas that are generated using the conductive nanoparticle ink on flexible substrates, such as Kapton and PET, are also reported in Refs.^9,12^. Despite these conductive nanoparticle inks can provide high conductivity, they can only be printed on specific substrates and carrier mediums^13^. For example, the self-sintering silver ink reported in Ref.^12^ can only achieve a low resistance with the use of commercial printing sheets. This will surely limit the compatibility of conductive nanoparticle ink since the radiation efficiency of the antenna is much affected by the dielectric loss of the substrate and the conductivity of the conductive ink trace^14^. Also, precise deposition of the conductive ink on the substrate requires a complex process, making the fabrication become a slow and non-scalable process^15^. A wearable antenna that was fabricated using electroplating thin metal foils such as copper and gold on elastic dielectric substrates was also reported in Refs.^7,8^. However, these antennas are not able to withstand tensile strain^16^. Therefore, there is a desire to have a compact, flexible, stable, conformal, and easy-to-make microwave antenna for wearable applications.

For microwave WPT, it involves a rectenna that has an antenna to receive the microwave power and a rectifier to convert it to DC power. Different types of rectennas have been reported, including those made of dipole antenna^17^, loop antenna^18^, patch antenna^11^, and fractal antenna^19^. Unfortunately, most of the reported rectennas, such as the one with an off-center fed dipole antenna (total length = 100 mm)^17^, a dual-band rectangular loop antenna (total size = 60 **×** 33 mm)^18^, and a microstrip patch antenna (total size = 70 **×** 70 mm)^11^, have a large footprint. In practice, to achieve better user experience, the wearable antennas are required to be compact, low profile, and lightweight^20^. However, very few of the reported solutions can meet the above-mentioned criteria. Besides that, a rectenna always requires the use of either impedance transforming/matching networks, resistance compression networks, or frequency selective networks, such as those reported in Refs.^21–23^, which will surely introduce additional losses and further increase the circuit complexity^11,23^. A compact fractal loop antenna reported in Ref.^19^ was incorporated with an in-loop ground plane (ILGP) so that it can provide sufficient impedance for matching with the rectifying circuit. However, again, it requires the use of a balun and multiple vias for the integration, which has increased the complexity of the circuit design process. Designing a compact and highly efficient 50 Ω rectenna that does not need a matching network remains a challenge for microwave WPT.

In this paper, a compact, conformal, and easy-to-make tattoo-polymer loop antenna is presented for designing a highly efficient rectenna. As human body is very lossy, it can cause the antenna’s radiation performance to deteriorate significantly. Degradation in the radiation pattern can result in transmission errors^24^. In our work, a tattoo-polymer 3 × 3 frequency selective surface (FSS) is integrated with the antenna to isolate it from the lossy human body. Both the antenna and FSS here can be rapidly and easily fabricated using the commercial tattoo paper and liquid metal alloy. The “Ag-In-Ga” traces in our antenna structure are coated with silver epoxy and liquid metal for achieving a higher conductivity (3.8 × 10^6^ S/cm). Our method has provided an alternative solution for the crucial problem frequently encountered by most fabric antennas, whose conductivities are usually low due to the limitation posed by embroidery density^5^. Different from the state-of-the-art rectennas^21–23^, here, a matching loop, a matching stub, and two coupled lines are employed for tuning the antenna impedance so that it can achieve a broad-band impedance matching with the rectifying circuit. The complexity of the antenna structure is simple as it does not require the use of any external matching networks. Finally, as our antenna is having 50 Ω impedance, it can integrate easily with the commercialized rectifiers, such as that demonstrated in Ref.^25^.

## Tattoo-polymer antenna design

### Antenna configuration

**Figure 1:**
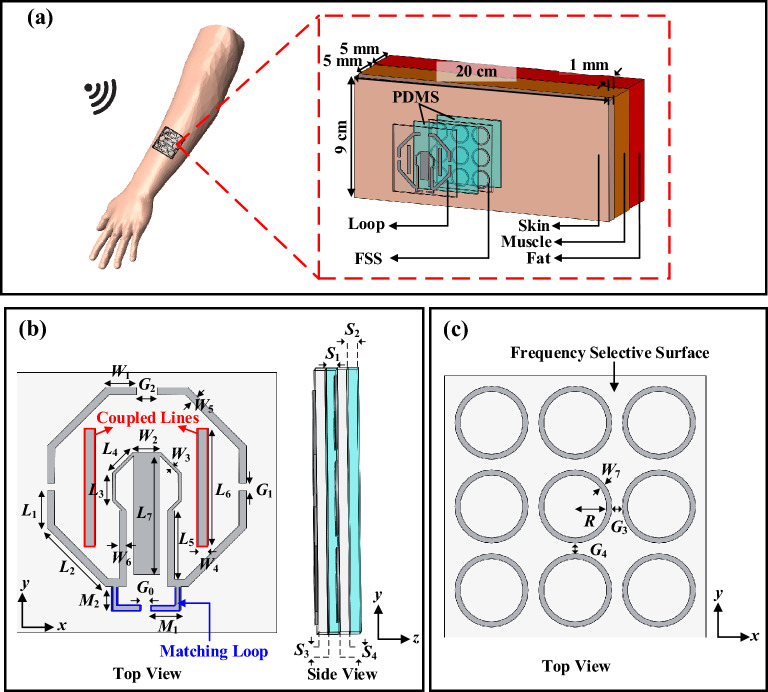
(**a**) Multilayer human tissue model composed of skin, fat, and muscle with an expanded view of the tattoo-polymer antenna. (**b**) Top and side views of the loop antenna. (**c**) Top view of the FSS.

In order to simulate the proposed tattoo-polymer antenna, we have employed a multilayer tissue model (Syndaver) comprising skin, fat, and muscle layers with an overall dimension of 20 × 9 × 11 mm^3^, as schematized in Fig. 1a, where the dielectric constants and loss tangents^26^ of all the layers are given in Supplementary Table S1. The proposed tattoo antenna consists of a planar octagonal loop antenna that operates at 2.40 GHz, which will be used for the on-body far-field WPT in the Wi-Fi range, as shown in Fig. 1b. Figure 1c shows the 3 × 3 ring-shaped FSS elements that have been used to isolate the antenna from human body. The fabrication processes of the proposed tattoo-polymer antenna and the FSS are described now. First, the antenna pattern is printed on a temporary tattoo paper (Amazing Raymond) with a thickness of 5 μm using an ordinary laser printer, followed by coating the printed traces using silver epoxy (MG Chemicals 8331S). Then, trace amount of eutectic Gallium–Indium (EGaIn) liquid metal is coated over the paper and cleaned using an aqueous solution (2 wt%) of acetic acid to remove the excess EGaIn. Prior to transferring to the human body, the tattoo-polymer antenna is covered with a thin transparent plastic through spray coating, which functions as an isolator, to avoid the impact from sweat, which can cause a reduction in the conductivity of the trace^13,14^. Finally, the tattoo antenna and the FSS were transferred to the PDMS substrate (*ε*_*r*_ = 2.5, and tan *δ* = 0.002) through the hydroprinting process. The PDMS is selected to be the structural support of the antenna due to its higher flexibility, lower cost, and easier fabrication, as compared with other polymer substrates^27^. The total profile of the proposed tattoo-polymer antenna is 2.70 mm, which is thinner than most of the state-of-the-art EBG/FSS-backed wearable antennas, as summarized in Supplementary Table S2^28–32^. Description of the detailed fabrication processes can be found in Refs.^13,15,33^. The final designs of the antenna and FSS are shown in Fig. 1a,b, with all the parameter values summarized in Table 1.

**Table 1:** List of design parameters.

Parameter	Dimension, mm	Parameter	Dimension, mm	Parameter	Dimension, mm
*L*_1_	4.23	*W*_1_	3.76	*G*_0_	2.00
*L*_2_	9.18	*W*_2_	3.83	*G*_1_	0.60
*L*_3_	3.83	*W*_3_	0.50	*G*_2_	1.55
*L*_4_	3.83	*W*_4_	1.00	*S*_*3*_	0.005
*L*_5_	7.14	*W*_5_	1.00	*S*_*4*_	0.005
*L*_6_	11.00	*W*_6_	1.00	*G*_4_	5.00
*L*_7_	14.65	*W*_7_	1.00	*G*_3_	5.00
*M*_1_	3.50	*S*_1_	0.50	*R*	5.00
*M*_2_	1.43	*S*_2_	2.20		

### Antenna design

**Figure 2:**
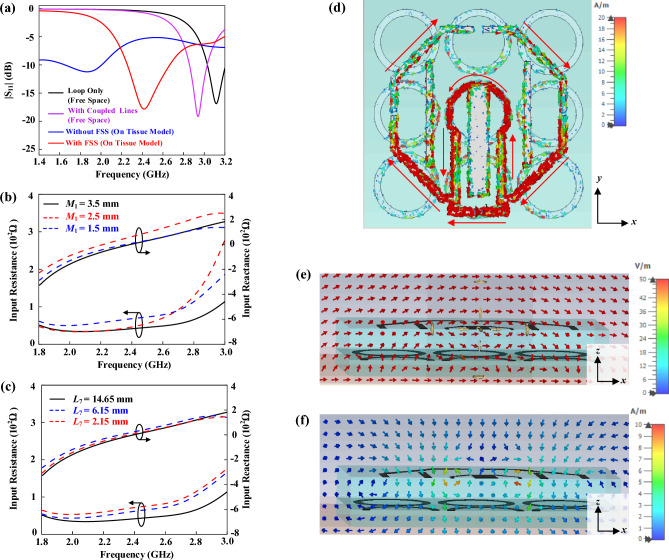
(**a**) Simulated |S_11_| of the proposed tattoo-polymer antenna. Effects of (**b**) the matching loop and (**c**) the matching stub on the antenna impedance. Simulated (**d**) current distribution, (**e**) E-field, and (**f**) H-field.

Although the loop antenna has been employed for harvesting energy^18,19^, it is usually very challenging to miniaturize its footprint as the circumference of a typical loop resonator is required to be in the multiple of its operating wavelength. Our proposed antenna is employed for designing a compact rectenna that can operate in the Wi-Fi range (2.40–2.48 GHz). Here, the octagonal loop is more preferred than the conventional square loop. This is because the 90° sharp bends of the square loop tend to generate stray radiation, and it can degrade the performance of the antenna^34^. To lower down its resonant frequency, here, two coupled lines are loaded to the loop antenna. A matching loop is appended to the loop for optimizing the antenna impedance, which will be discussed in detail shortly. The design procedure starts with simulating the reflection coefficient of a simple octagonal loop without the two coupled lines in free space. With reference to Fig. 2a, the dominant mode is found to be located at 3.12 GHz (or *λ* = 96.15 mm), which corresponds well to the perimeter of one circumference (101.15 mm). With the inclusion of the two coupled lines (*L*_6_ = 11.00 mm), as can be seen from the same figure, the resonant frequency has shifted down to 2.94 GHz. After performing the simulation in free space, which can be done in a faster manner, the antenna is now attached to the human tissue model for further optimization. Due to the loading effect of the high-dielectric lossy tissue^35^, the resonant frequency of the antenna has further shifted down to 1.86 GHz, which has caused poor impedance matching. It shows that the tissue layer has deteriorated the impedance performance of the antenna significantly due to its high loss. Then, a 3 × 3 FSS layer is inserted in between the loop resonator and the human tissue as an isolator. With the inclusion of the FSS and PDMS substrates, the antenna is fine-tuned so that it operates at 2.40 GHz with good impedance matching, as shown in Fig. 2a. By comparing with the rectangular loop (circumference = 120.30 mm, 0.96*λ*_2.4GHz_) in Ref.^18^ and the fractal loop (circumference = 215.50 mm, 1.30*λ*_1.8 GHz_) in Ref.^19^, the circumference of our octagonal loop antenna (94.60 mm, 0.76*λ*_2.4 GHz_) is 21.8% and 56.1% smaller than the former and the latter, indicating that ours is more compact.

Since the antenna is to be used for WPT, it is very important for the tattoo-polymer antenna to have a good impedance matching with the RF-to-DC rectifier circuit for maximizing the RF power transfer. Although matching circuits and filtering networks can be introduced for improving the impedance matching, they can increase the complexity and size of the antenna system^36^. To solve this problem, our antenna is integrated with a matching loop, a matching stub, and two coupled lines for tuning the antenna impedance for matching with the 50 Ω rectifier. By tuning the lengths of the matching loop (*M*_1_) and the matching stub (*L*_7_), good impedance matching can be easily realized over a wide bandwidth, as shown in Fig. 2b,c. Figure 2d shows the surface current distribution on the final tattoo-polymer antenna, with the corresponding electric and magnetic fields given in Fig. 2e,f. Currents are found to be denser near to the feeding port. Due to the small open gap on the opposite end, the current intensity diminishes at this location. Strong currents on the matching loop show that it is highly inductive, implying that tuning the loop is effective for changing the antenna impedance. It can also be justified from the electric and magnetic fields that the loop antenna has radiated well. Also observed is that the FSS layer has insulated the antenna from the human tissue effectively.

### FSS design and SAR evaluation

**Figure 3:**
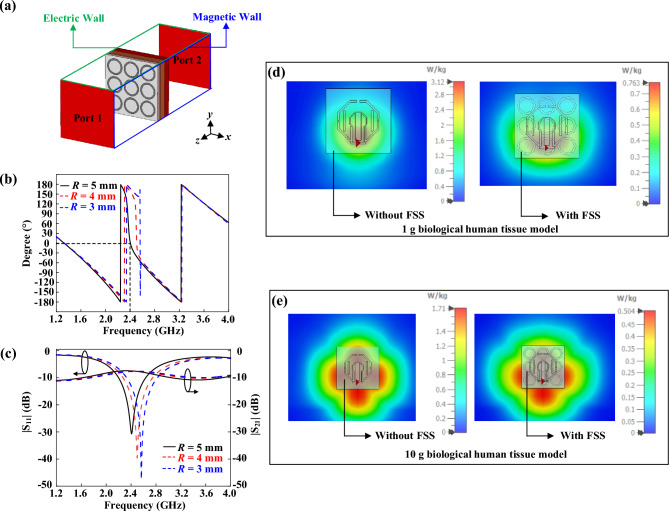
(**a**) FSS model inside a unit cell. (**b**) Reflection phase for different element radii. (**c**) Simulated reflection and transmission coefficients. Simulated SAR value for (**d**) 1 g biological tissue without and with FSS, and (**e**) 10 g biological tissue without and with FSS.

In this section, the design procedure of the FSS is discussed. The ring-shaped FSS is selected because it has a simple structure. According to a study presented in Ref.^37^, the ring FSS has low back radiation when it is integrated with a dipole. First, the FSS element is placed on the human tissue model and simulated inside a unit cell for finite structure, which is available in the CST MWS software, as shown in Fig. 3a. Two waveguide ports are employed to generate propagating plane waves in the + *z* and − *z* directions. With reference to the same figure, two magnetic walls (*H*_*t*_ = 0) are set in perpendicular to the *x* direction, while two electric walls (*E*_*t*_ = 0) are vertically defined in the *y* direction. Figure 3b shows the simulated reflection phase of the ring-shaped FSS element with different radii [*R* = 5 mm (0.042*λ*_2.4GHz_), 4 mm (0.032*λ*_2.4GHz_), and 3 mm (0.025*λ*_2.4GHz_)] in the phase range of 180° to *−* 180*°* with respect to frequency. The reflection phase is obtained by de-embedding the reference plane to the surface of the element. A reflection phase of ~ 0° is observed near to the operating frequency of 2.40 GHz^38^ for the case *R* = 0.025*λ*_2.4GHz_. This is to make sure that the reflected wave is in phase with the incident wave on the tissue model^39^. The corresponding transmission and reflection coefficients are also simulated and shown in Fig. 3c. For the case of *R* = 0.025*λ*_2.4GHz_, the reflection and transmission coefficients are found to be − 30.7 dB and − 7.45 dB, respectively, which correspond to 0.029 and 0.424 at the operating frequency of 2.40 GHz. For the calculated insertion loss 1 − |S_11_|^2^ − |S_22_|^2^ = 0.82, it is observed that the human tissue is extremely lossy due to its high insertion loss.

Since the proposed tattoo-polymer antenna is designed for wearable applications, the specific absorption rate (SAR) is an important factor to be considered as it is required to be placed near to human body^35^. Here, the SAR simulation is conducted by establishing a multilayer biological human tissue model using the CST software. Then, the tattoo-polymer antenna is placed right above the biological tissue with a separation distance of 5 mm^28^. The input power to the antenna is set to be 100 mW at 2.40 GHz, and the SAR is calculated according to the IEEE C 95.1 standards. The SAR values are simulated by averaging over the sampled volumes of 1 g and 10 g. By comparing Fig. 3d, it is observed that the FSS layer has successfully reduced the SAR from 3.12 W/kg to 0.76 W/kg. Similar trend is also observed in Fig. 3e, where the corresponding SAR can be cut down from 1.71 to 0.50 W/kg. A comparison has been drawn in Table 2. The FSS layer is found to have reduced the SAR by as much as 75.5% and 70.0%, respectively, for the biological tissues at the sampled volumes of 1 g and 10 g. By comparing with the liquid EBG-backed stretchable slot antenna in Ref.^40^ and the EBG-backed planar inverted-F antenna in Ref.^41^, the SARs of our FSS-integrated octagonal loop antenna are 64.0% and 27.6% lower than those in Refs.^40,41^ by referencing the volume of 1 g tissue sample (USA Standards); while they are 34% and 42% lower than the reported wearable antennas in Refs.^41,42^ with 10 g tissue sample volume (European Standards), indicating that our FSS-integrated octagonal loop antenna has achieved a lower SAR with a more compact size. The details of the comparison are summarized in Supplementary Table S3.

**Table 2:** Simulated SAR value.

Sample Volume, g	Antenna Alone	With FSS	Reduction, %
1	3.12	0.76	75.5
10	1.71	0.50	70.0

## Results

### Antenna radiation characterization

**Figure 4:**
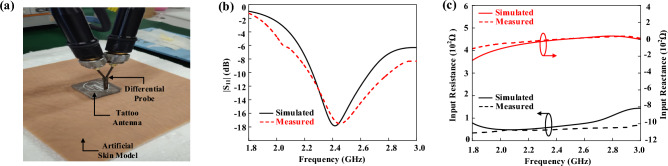
(**a**) Measurement experimental setup for reflection coefficients. Simulated and measured (**b**) reflection coefficients and (**c**) antenna impedances.

The tattoo-polymer antenna was integrated with the FSS and fabricated, and experiment was carried out using a differential probe^43^, as depicted in Fig. 4a. Here, the antenna is placed on a medical artificial skin layer^26^. Figure 4b illustrates the simulated and measured reflection coefficients of the FSS-integrated tattoo-polymer antenna, where good agreement is observed between the simulation and measurement. It implies that the multilayer human tissue model can be used to represent the properties of the artificial skin layer very well. With reference to the figure, the measured resonant frequency is ~ 0.05 GHz higher than the simulated one, with a discrepancy of ~ 2%, which can be caused by the fabrication tolerances of the tattoo-polymer antenna. The corresponding antenna impedances are demonstrated in Fig. 4c, showing reasonable agreement (discrepancy of ~ 2% at 2.45 GHz resonant frequency) between simulation and experiment.

Supplementary Figure S1 shows the simulated and measured far-field radiation patterns (*yz*-plane, *xz*-plane, and *xy*-plane) of the integrated tattoo-polymer at 2.40 GHz when it is placed on the artificial skin layer. For comparison purpose, the radiation patterns of the loop antenna on tissue without FSS (as shown in Supplementary Fig. S1) are also analyzed at the same frequency. The measured antenna gain of the proposed FSS-integrated tattoo-polymer is − 2.33 dB (simulation − 1.13 dB) at *θ* = 0°, which is 16.14 dB higher than that for the case without any FSS (simulation − 15.70 dB). A considerable gain improvement (13.8 dB) in the boresight can be achieved by integrating the loop antenna with the FSS structure. It shows that the FSS layer has successfully isolated the antenna from the skin, and it has improved the radiation performance significantly.

### Deformation and loading effects

**Figure 5:**
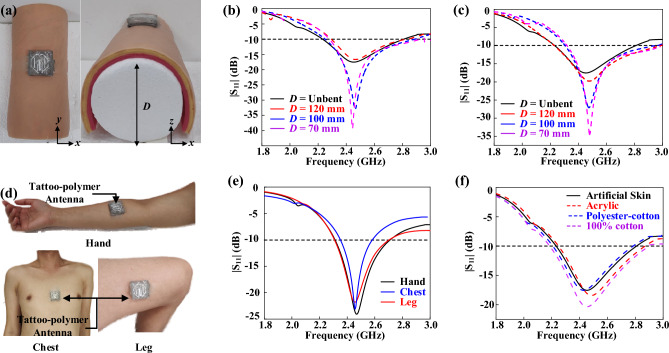
(**a**) Experimental setup for bending the FSS-integrated tattoo-polymer antenna along the *x*-direction. Measured reflection coefficient when it is bent along (**b**) *y*-axis and (**c**) *x*-axis. FSS-integrated antenna placed on different body locations: (**a**) Hand, (**b**) chest, (**c**) and leg. Measured reflection coefficient on acrylic plate and fabrics.

In practical applications, a wearable antenna needs to be worn on the human body, which may result in bending or deformation. The resonant frequency and impedance matching of the antenna are susceptible to changes due to structural deformation^38^. Therefore, it is crucial to ensure that the operating frequency of a wearable antenna remains stable even when it undergoes deformation. An experiment was performed based on the structure shown in Fig. 5a, and the detailed setup can be found in the methods section. The resonant frequencies of the tattoo-polymer antenna for different bending scenarios in the x- and y-axis were measured and plotted in Fig. 5b,c. In all cases, the resonant frequency and impedance bandwidth (|S11| < − 0 dB, ⁓ 24% fractional bandwidth with and without bending) were found to be relatively stable and not significantly affected by the bending curvature. This is an important characteristic for wearable electronics.

The impact of the backing objects on the antenna performance was also investigated in practical scenarios. The FSS-integrated tattoo-polymer antenna was placed on hand, chest, and leg of a male volunteer as depicted in Fig. 5d. The reflection coefficient (|S_11_|) was measured in Fig. 5e to study the effects of different parts of the human body on the tattoo-polymer antenna. The resonant frequency of the FSS-integrated tattoo-polymer was measured to be 2.44 GHz when it is placed on leg and chest with fractional bandwidths of 16.0% and 10%, respectively. A slight shift (0.02 GHz) was noted when the antenna is attached on the hand, where the resonant frequency is now 2.47 GHz with a fractional bandwidth of 16.0%. For all the cases, the FSS layer has successfully shielded the antenna from the human body, although the fractional bandwidth can be slightly affected. The FSS-integrated tattoo-polymer was also tested on the common wearable materials such as fabrics [100% cotton and polyester-cotton (65% cotton and 35% polyester)] and acrylic plate for simulating the real-life scenarios (clothing and watches). The measurement results are shown in Fig. 5f. For ease of comparison, the measured reflection coefficient for the artificial skin is also plotted in the same figure. With reference to Fig. 5f, the resonant frequency shifts upward towards 2.48 GHz (fractional bandwidth = 24.5%) when the antenna is placed on acrylic. The frequency has slightly decreased to 2.43 GHz (fractional bandwidth = 24.0%) and 2.44 GHz (fractional bandwidth = 28.0%) when the antenna is placed on the polyester-cotton and 100% cotton, respectively. In conclusion, the loading fabrics have very little effect on the fractional bandwidth and resonant frequency of the FSS-integrated tattoo-polymer, which is a very desired feature for the wearable electronics.

### Wireless power transfer evaluation

**Figure 6:**
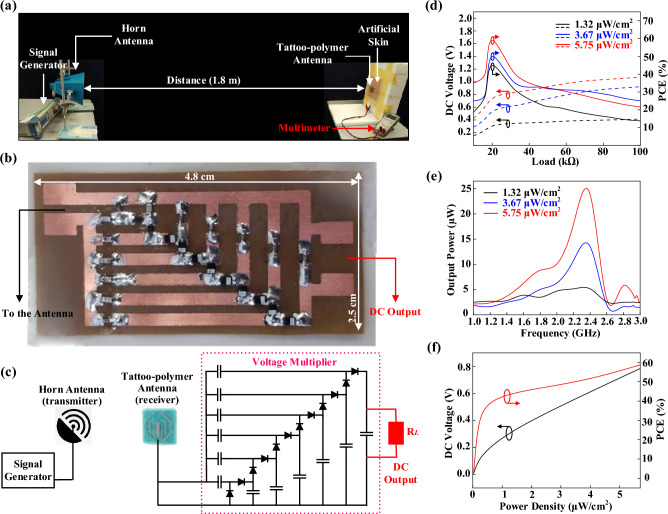
(**a**) Wireless power measurement setup. (**b**) 6-stage voltage multiplier using BAT15-03W diode (50 Ω impedance). (**c**) Schematic diagram of the 6-stage voltage multiplier. (**d**) Measured wireless DC output of the antenna at 2.45 GHz under load sweep. (**e**) Measured wireless DC output power of the antenna with the optimal load 20 kΩ load at different frequencies. (**f**) Measured wireless DC voltage output and PCE of the antenna under power sweep with the optimal load 20 kΩ.

**Figure 7:**
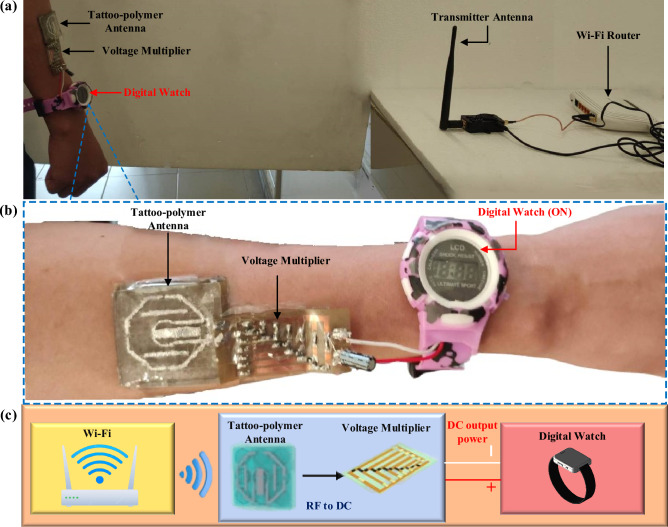
(**a**) Demonstration of on-body WPT using a Wi-Fi router and the proposed rectenna. (**b**) Closeup view of the proposed rectenna and a digital watch. (**c**) Block diagram of the on-body WPT using a Wi-Fi router and the proposed rectenna.

**Figure 8:**
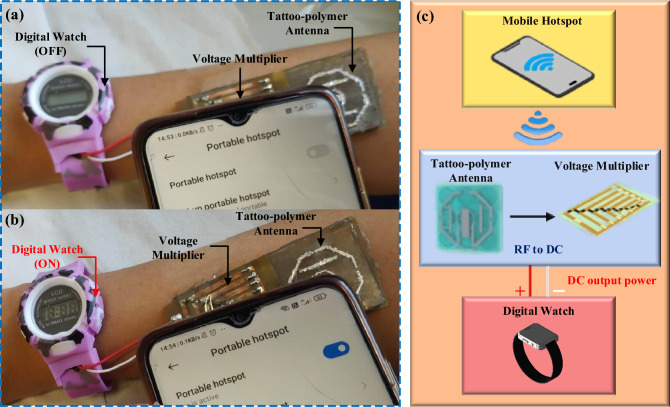
Demonstration of on-body WPT using a mobile hotspot and the proposed rectenna: (**a**) Mobile hotspot is under OFF condition, (**b**) mobile hotspot is under ON condition. (**c**) Block diagram of the on-body WPT using a mobile hotspot and the proposed rectenna.

The wireless power performance of the proposed tattoo-polymer antenna was evaluated by conducting a load sweep from 0 to 100 kΩ at three fixed power densities, *S* (5.75, 3.67, and 1.32 μW/cm^2^) at 2.45 GHz, using the experimental setup depicted in Fig 6a. The output voltage from the tattoo-polymer antenna was converted into DC using the circuit shown in Fig. 6b. Further details on the measurement setup can be found in the methods section. Figure 6c shows the proposed rectenna can achieve a maximum PCE of 59.0%, 49.8%, and 44.7%, respectively, with 0.72, 0.59, and 0.33 V_DC_ over a 20 kΩ load. It is observed that the output voltage of the proposed antenna is linear, and the optimal load does not vary with different power densities. It implies that the proposed design does not require the maximum power point tracking (MPPT) capability to maintain its maximum PCE^11^, which is a very desirable feature. After selecting the optimal load (20 kΩ), a frequency sweep is performed from 1.0 and 3.0 GHz at different power densities (*S*) for measuring the bandwidth of the antenna. Figure 6d shows the output power of the rectifying tattoo-polymer antenna at different frequencies, showing that this rectenna is optimum within the entire passband (⁓ 2.5% covering 2.2–2.5 GHz). Lastly, a power sweep is carried out to study the antenna’s performance at different *S*. Figure 6e shows the measured DC output of the rectenna for 0*.*1 < *S* < 5.5 μW/cm^2^ with the optimal load (20 kΩ). It is observed that the PCE of the rectenna exceeds 40% from 1.0 μW/cm^2^ onwards, indicating that it is suitable to be used for low power levels as well. As proof of concept, a commercialized digital watch was connected to the output of the proposed rectenna, as shown in Figs. 7a and 8a. Here, a 2.40 GHz Wi-Fi router (Tp-link TL-WR841N) and a mobile hotspot were adopted as the microwave energy sources for the proposed rectenna. With reference to the closeup view in Fig. 7b, the digital watch was successfully powered up, which indicates successful WPT in between the proposed rectenna and the Wi-Fi router. Moreover, Fig. 8a,b show that the digital watch was also successfully powered up using the mobile hotspot. The corresponding block diagrams are presented in Figs. 7c and 8c. The demonstrations have shown the potential applications of the proposed tattoo-polymer antenna for on-body WPT.

## Discussion

The proposed rectenna is compared with the state-of-the-art low-power rectennas, as shown in Supplementary Table S4. Our rectenna can achieve a higher PCE than those textile antennas in Refs.^38,44^. This is because our antenna does not need the use of external matching circuits and long transmission lines, which can introduce additional insertion loss. With reference to the table, similar level of PCE was achievable for the wearable rectennas in Refs.^11,19^. A resistance compress matching network was employed in Ref.^11^ so that a higher DC voltage sensitivity and a better PCE could be achieved. While in Ref.^19^, an in-loop ground plane (ILGP) was engaged to adjust a loop antenna’s impedance to ⁓ 50 Ω for attaining a high PCE (61%). The ILGP structure was connected to the loop antenna through a metallic via. For both, however, the employment of these additional external circuits will surely increase the antenna complexity. In contrast, our proposed antenna structure is rather simple as it does not require the use of any external matching networks. Besides that, many other rectennas, which are proposed for WPT and energy harvesting (EH), have put emphasis on maximizing the PCE. However, high PCE was often achievable only at high power density levels (more than 100 μW/cm^2^)^45^. For example, Falkenstein et al. reported a dual linearly polarized patch antenna with total efficiencies over 50% for a high power density of 25–200 μW/cm^2^^46^. Harouni et al. reported a 2.45 GHz compact dual-circularly polarized rectenna with PCE = 63% for a high power density of 525 μW/cm^2^^47^. Also, high PCEs of 84.4% and 82.7% at 2.45 and 5.80 GHz, respectively, were achieved by the CPS dipole dual-frequency antenna reported by Suh^48^. Although the reported rectennas have reasonably better PCE at a higher power density, practically, the RF/microwave power available in an ambient environment is usually very low. For example, based on the London RF survey measurement reported by Pinuela^49^, the average power density of the Wi-Fi (2.40–2.50 GHz) is only 0.89 nW/cm^2^ in most of the underground stations. The power density was found to be in the range of 0.0017–0.8594 μW/cm^2^ for the European Union (EU) telecommunication spectrum of 10 MHz–6 GHz under long-term RF EMF measurements^50^. Therefore, it is important that a rectenna can operate and maintain a high efficiency under an ultralow power density. By comparing with the reported rectennas in Refs.^45,50^, our proposed rectenna can achieve higher efficiencies of 8.5% and 7.5%, respectively, with lower power densities of 125% and 110%, indicating that our proposed rectenna can achieve a higher sensitivity.

In summary, this work has presented the design and measurement of a compact tattoo-polymer FSS-integrated loop antenna for on-body wireless power transfer with high PCE, which is operating well under low power levels, and it is intrinsically matched to 50 Ω without needing any external matching circuits. Proof of concept demonstration shows that WPT can be achieved through the proposed rectenna by harvesting energies from the microwave/RF sources such as the Wi-Fi router and mobile hotspot. Recent advances in the ultralow-power electronics have led to several reported wearable devices with power consumption as low as nW to μW, such as a pressure sensor (minimum power consumption = 10.0 μW)^51^, an organic pulse oximetry sensor (minimum power consumption = 24.0 μW)^52^, and a biomedical electrocardiogram (minimum power consumption = 75.0 nW)^53^. Based on the results shown in Fig. 6, the proposed tattoo-polymer antenna can achieve a maximum output power of ~ 26.0 μW, and this power is sufficient for these recently reported ultralow-powered wearable devices. Also, with the rise of 5G technology, beamforming techniques are massively used, and this has made the microwave WPT an advantageous power source^4^. Although the prospect is promising, the application of the tattoo-polymer antenna to microwave WPT is still in the nascent stages. Many crucial issues, such as wear resistance and long-term durability, are yet to be explored. Further improvements on the rectenna’s maximum output power and PCE are always needed to power up a wider range of electronic devices with a power consumption in the range of mW. Therefore, future efforts will focus on further characterization of the device durability and WPT output power of the proposed tattoo-polymer antenna, which includes encapsulation with a Tegaderm film to improve the wear resistance as well as enhancement in the output power through a commercialized booster module or array unit.

## Conclusion

A wearable tattoo-polymer FSS-integrated loop antenna has been proposed for wireless power transfer. Both the antenna and FSS can be easily printed on a piece of tattoo paper. The footprint and profile of the proposed antenna are smaller than most of the state-of-the-art wearable antennas. The FSS layer has successfully improved the radiation performance of the loop antenna on human body in the boresight, with a significant gain improvement of ~ 13.8 dB. Also, the FSS can improve the SAR as much as 75.5% on the biological tissue. Bending test has been performed on the proposed wearable antenna, where the resonant frequency and impedance bandwidth are found to be very stable. The antenna performance is not affected much by its own structural deformation. For wireless power transfer, the FSS-integrated antenna was bonded with a 50 Ω 6-stage voltage multiplier. It has been observed from experiments that a maximum efficiency of 59.0% is achievable with an input power of 5.75 μW/cm^2^. The efficiency is found to be able to exceed 40% even for a low input power of 1.0 μW/cm^2^. It shows that our proposed rectenna can operate well under low power levels.

## Method

### Antenna deformation and loading effect measurement

The proposed FSS-integrated tattoo-polymer was placed on a man-made curvature created by wrapping artificial skin around cylindrical polystyrene foam of diameters 70 mm, 100 mm, and 120 mm to produce various degrees of structural deformation as depicted in Fig. 5a. The antenna's bending in the x- and y-axis can be measured by rotating it 90° around its own z-axis. The chosen diameters simulate the typical sizes of a human leg, hand, and chest^28^. The effects of backing loading objects are analyzed for realistic scenarios. The FSS-integrated tattoo-polymer was placed on the hand, chest, and leg, as shown in Fig. 5d. It was also tested on common wearable materials such as cotton and polyester-cotton fabrics and acrylic plates to simulate real-life conditions (clothing and watches). The on-body measurement was approved by Universiti Tunku Abdul Rahman under its Research Ethics & Code of Conduct (POL IPSR R&D 004) and Code of Practice for Research Involving Humans (POL IPSR R&D 005), and was expedited reviewed by the Institute of Postgraduate Studies and Research (IPSR) at Universiti Tunku Abdul Rahman. Informed consent was obtained from participants. All experiments were conducted following relevant guidelines and regulations.

### Far-field wireless power transfer measurement

A microwave signal generator (Rohde & Schwarz SMB100A) was utilized to transmit a continuous wave at 2.45 GHz (with power ranging from 0 to 30 dBm) using a horn antenna as the source. The tattoo-polymer antenna, placed 1.80 m away from the source, was connected to a variable resistive load through its DC output. To meet the minimum Fraunhofer far-field criteria, a distance of 1.20 m was maintained between the horn antenna and the proposed antenna. A 6-stage voltage multiplier, designed using BAT15-03W diodes (low barrier Schottky diodes), was utilized to convert the harvested microwave energy into a DC voltage. The rectifier circuit, fabricated on a flexible PCB, was modified from Ref.^44^ to match the antenna impedance of 50 Ω. Fig. 6 illustrates the overall experimental setup and the 6-stage voltage multiplier. The available power at the receiving tattoo-polymer antenna can be calculated from the power density of the incident plane wave and the antenna's effective area (*A*_*eff*_)^47^, as indicated by Eq. (1)

1 $$A_{eff} { = }G_{R} {\frac{{\lambda^{{2}} }}{4\pi }},$$

where GR represents the gain of the tattoo-polymer antenna at the receiving end. The power conversion efficiency (PCE) of the receiving antenna can be calculated using Eq. (2) ^48^.

2 $${\text{PCE = }}{\frac{{V^{{2}} }}{{Z_{L} SA_{eff} }}},$$

where *V* is the measured output DC voltage across a load *Z*_*L*_, and *S* is the power density of the incident plane wave.

## Supplementary Information

Supplementary Information.

## Data Availability

The datasets used and analysed during the current study available from the corresponding author on reasonable request.
