# Supplementary material for: Compact conformal tattoo-polymer antenna for on-body wireless power transfer
Source: Sci Rep. 2023 Jun 15;13:9678. doi: 10.1038/s41598-023-36335-6 (PMC10272206; doi:10.1038/s41598-023-36335-6)
Supplement: Supplementary file 1 — Supplementary Information. [file 41598_2023_36335_MOESM1_ESM.docx]

**Supplementary Information**

**Compact Conformal Tattoo-Polymer Antenna for On-body Wireless Power Transfer**

# Xi Liang Chang1, Pei Song Chee1,*, and Eng Hock Lim2,*

1Department of Mechatronics and Biomedical Engineering, Universiti Tunku Abdul Rahman, Kajang, 43000, Malaysia

2Department of Electrical and Electronic Engineering, Universiti Tunku Abdul Rahman, Kajang, 43000, Malaysia

* cheeps@utar.edu.my

* limeh@utar.edu.my

**Supplementary Table S1.** Dielectric parameters of the human tissue model

| **Layer** | **Dielectric Constant, *ε_r_*** | **Loss Tangent, tan *δ*** |
| --- | --- | --- |
| Skin | 37.95 | 0.2835 |
| Fat | 5.27 | 0.1 |
| Muscle | 52.67 | 0.2835 |

**Supplementary Table S2.** Comparison with other state-of-the-art EBG/FSS-backed wearable antennas.

| **Ref** | **Overall Antenna Size, λ^2^** | **Overall Profile, mm** | **Frequency, GHz** |
| --- | --- | --- | --- |
| [51] | 0.32 × 0.26 | 4.00 | 2.40 |
| [34] | 0.37 × 0.29 | 5.50 | 2.45 |
| [52] | 1.52 × 1.20 | 5.10 | 24.0 |
| [53] | 1.01 × 0.74 | 3.00 | 2.45 |
| [54] | 0.82 × 0.82 | 6.00 | 2.45 |
| **This Work** | **0.28 × 0.28** | **2.70** | **2.42** |

# References

34. Gao, G., Wang, S., Zhang, R., Yang, C., & Hu, B. Flexible EBG‐backed PIFA based on conductive textile and PDMS for wearable applications*. Microwave and Optical Tech. Lett.* **62**, 1733-1741 (2020).

51. Gao, G., Zhang, R., Yang, C., Meng, H., Geng, W. & Hu, B. Microstrip monopole antenna with a novel UC-EBG for 2.4 GHz WBAN applications. *IET Microwaves, Antennas & Propag*. **13**, 2319-2323 (2019).

52. Iqbal, A., Basir, A., Smida, A., Mallat, N. K., Elfergani, I., Rodriguez, J., & Kim, S. Electromagnetic bandgap backed millimeter-wave MIMO antenna for wearable applications. IEEE Access. **7**, 111135-111144 (2019).

53. Velan, S. et al. Dual-band EBG integrated monopole antenna deploying fractal geometry for wearable applications. *IEEE Antennas Wirel. Propag. Lett*. **14**, 249-252, (2014).

54. Lago, H., Soh, P. J., Jamlos, M. F., Shohaimi, N., Yan, S. & Vandenbosch, G. A. Textile antenna integrated with compact AMC and parasitic elements for WLAN/WBAN application. *Applied Phy. A*. **122**, 1-6 (2016).

**Supplementary Table S3.** SAR comparison with other state-of-the-art EBG/FSS/AMC-backed wearable antennas.

| **Ref** | **Frequency, GHz** | **Antenna Size (included the size of metasurface), mm^3^** | **Sample Volume, g** | **SAR, W/kg** |
| --- | --- | --- | --- | --- |
| [35] | 3.40 | 69.5 × 33.0 × 14.0 | 1  10 | 1.25  0.45 |
| [36] | 2.45 | 81.0 × 81.0 × 4.0 | 1  10 | 0.97  0.67 |
| [37] | 2.45 | 50.0 × 50.0 × 9.5 | 1  10 | -  0.71 |
| **This work** | **2.45** | **35.0 × 35.0 × 2.7** | **1**  **10** | **0.76**  **0.50** |

# References

35. Low, J. H., Chee, P. S., & Lim, E. H. Liquid EBG-backed stretchable slot antenna for human body. *IEEE Trans. Antennas Propag* **70**, 9120-9129 (2022).

36. Gao, G., Hu, B., Wang, S., & Yang, C. Wearable planar inverted‐F antenna with stable characteristic and low specific absorption rate. *Micro. and Optical Tech. Lett*. **60**, 876-882 (2018).

37. Agarwal, K., Guo, Y. X., & Salam, B. Wearable AMC backed near-endfire antenna for on-body communications on latex substrate. *IEEE Trans. comp., pack. manufac. tech*. **6**, 346-358 (2016).

**Supplementary Figure S1.** Simulated and measured far-field radiation patterns for the cases with and without FSS on (a) *yz*-plane, (b) *xz*-plane, and (c) *xy*-plane.

**Supplementary Table S4.** Comparison with the state-of-the-art rectennas.

| **Ref.** | **Frequency, GHz** | **Power Density, *μ*W/cm^2^** | **Maximum PCE, %** | **Substrate** |
| --- | --- | --- | --- | --- |
| [40] | 0.87 | 14.0 | 50 | Textile |
| [41] | 6.80 | 70.8 | 26.7 | Textile |
| [19] | 1.80 | 10.0 | 61.0 | PCB (rigid) |
| [11] | 2.45 | 200 | 62.0 | Textile |
| [42] | 0.86 | 2.1 | 37 | PCB (rigid) |
| [47] | 0.86 | 2.25 | 36.6 | Wire |
| **This Work** | **2.45** | **5.75**  **1.0** | **59.0**  **40.2** | **Tattoo Paper + PDMS Substrate** |

# References

# Adami, S. E. *et al*. A flexible 2.45 GHz power harvesting wristband with net system output from− 24.3 dBm of RF power. *IEEE Trans. Microwave Theory Tech*. 66, 380-395 (2017).

# Zeng, M., Andrenko, A. S., Liu, X., Li, Z., & Tan, H. Z. A compact fractal loop rectenna for RF energy harvesting. *IEEE Antennas Wirel. Propag. Lett*. 16, 2424-2427 (2017).

1. Monti, G., Corchia, L., & Tarricone, L. UHF wearable rectenna on textile materials," *IEEE Trans. Antennas Propag.* **61**, pp. 3869-3873, (2013).
2. Naresh, B., Singh, V. K., & Bhargavi, V. Low power circularly polarized wearable Rectenna for RF energy harvesting. *Adv. Power Sys. Energy Management: Springer*. 131-138 (2018).
3. Okba, A., Takacs, A., & Aubert, H. Compact rectennas for ultra-low-power wireless transmission applications. *IEEE Antennas Wirel. Propag. Lett.* **67**, 1697-1707 (2019).
4. Assimonis, S. D., *et al*. Efficient and sensitive electrically small rectenna for ultra-low power RF energy harvesting. *Sci. reports*. **8**, 1-13 (2018).
